# The Comprehensive Snack Parenting Questionnaire (CSPQ): Development and Test-Retest Reliability

**DOI:** 10.3390/ijerph15050862

**Published:** 2018-04-26

**Authors:** Dorus W. M. Gevers, Stef P. J. Kremers, Nanne K. de Vries, Patricia van Assema

**Affiliations:** 1 Department of Health Promotion, NUTRIM School for Nutrition and Translational Research in Metabolism, Maastricht University Medical Centre+, P.O. Box 616, 6200 Maastricht, The Netherlands; s.kremers@maastrichtuniversity.nl (S.P.J.K.); n.devries@maastrichtuniversity.nl (N.K.d.V.); p.vanassema@maastrichtuniversity.nl (P.v.A.); 2 Department of Health Promotion, CAPHRI School for Public Health and Primary Care, Maastricht University Medical Centre+, P.O. Box 616, 6200 Maastricht, The Netherlands

**Keywords:** food parenting practices, questionnaire, reliability, snack intake, children, CSPQ, snacking

## Abstract

The narrow focus of existing food parenting instruments led us to develop a food parenting practices instrument measuring the full range of food practices constructs with a focus on snacking behavior. We present the development of the questionnaire and our research on the test-retest reliability. The developed Comprehensive Snack Parenting Questionnaire (CSPQ) covers 21 constructs. Test-retest reliability was assessed by calculating intra class correlation coefficients and percentage agreement after two administrations of the CSPQ among a sample of 66 Dutch parents. Test-retest reliability analysis revealed acceptable intra class correlation coefficients (≥0.41) or agreement scores (≥0.60) for all items. These results, together with earlier work, suggest sufficient psychometric characteristics. The comprehensive, but brief CSPQ opens up chances for highly essential but unstudied research questions to understand and predict children’s snack intake. Example applications include studying the interactional nature of food parenting practices or interactions of food parenting with general parenting or child characteristics.

## 1. Introduction

Childhood overweight and obesity have become widespread and are a great burden to both the individual involved and society [1,2]. Evidence indicates that overweight children have an increased risk to become overweight in adulthood [3] and that dietary patterns acquired in childhood track into adulthood [4]. Parental behaviors seem to be important in this respect [5]. Behaviors that may affect children’s dietary pattern may be called Food Parenting Practices (FPPs) [6], and include for example restricting the child’s access to foods or pressuring the child to eat certain types of food [7]. Many of them, particularly coercive or controlling types, likely occur between meals [8]. Intakes of energy-dense snack foods (EDSFs) can highly contribute to a child’s daily energy intake [9]. Based on national food consumption survey data, Dutch children consume about 1600 kJ (about 375 kcal) EDSFs per day on average, excluding drinks [10].

The evidence about the impact of many parenting practices on children’s snack intake is inconclusive and incomplete [11]. Likely, two issues explain this lack of an unambiguous and complete evidence base. First, existing instruments contain inconsistencies: similar constructs are given different labels and are operationalized inconsistently across instruments [12]. Second, many existing instruments are operationalized in a very narrow fashion (e.g., only assessing controlling practices such as restriction or pressure to eat) [13]. Therefore, there is less evidence on the desirability of other practices, compared to controlling practices. In 2012, a conference on the measurement of parenting was set up which served as a starting point for several initiatives to solve these issues [6]. Our Delphi study [14], which identified and defined food parenting constructs around snacking, was one of them. The next step was to convert identified constructs into instruments.

We aimed to systematically develop a concise instrument measuring the full range of FPP constructs around snacking and targeted Dutch parents of children aged 4–12 years old. The current article presents an overview of the development of the Comprehensive Snack Parenting questionnaire (CSPQ) and an assessment its test-retest reliability.

## 2. Materials and Methods

**Figure 1:**
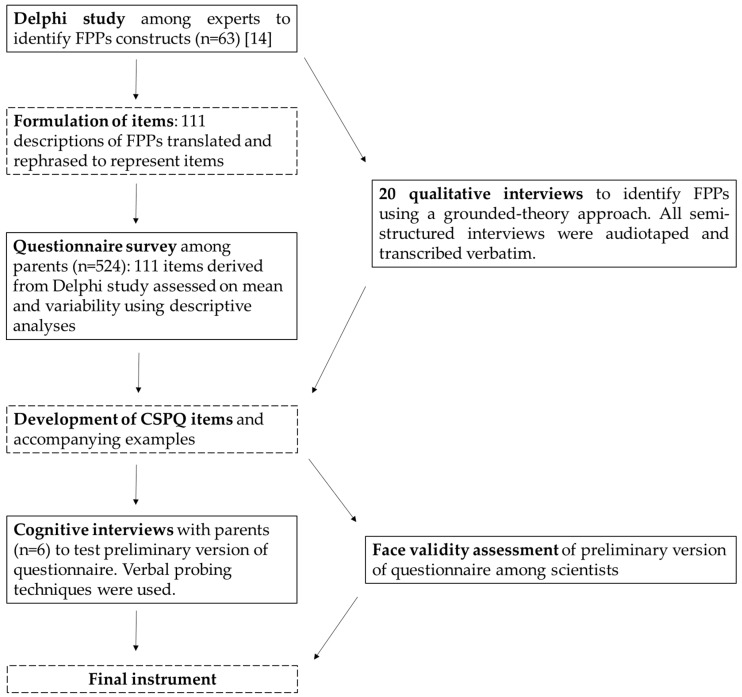
Flow chart of the Comprehensive Snack Parenting Questionnaire (CSPQ) development.

The CSPQ containing 21 items was developed through a systematic combination of methods (see Figure 1) for: (1) identification of food parenting practices (i.e., expert consultation through the previous described Delphi study [14] and qualitative interviews with parents); (2) item development (i.e., the Delphi study, the qualitative interviews with parents, and a pilot questionnaire survey); and (3) refinement of the instrument (i.e., cognitive interviews with parents and expert reviews).

The final instrument was administered to a group of parents. Parents were invited to fill out the questionnaire twice, to assess test-retest reliability. Each of the steps in the development and reliability assessment process is elaborated below. According to the Dutch law, review by an ethics committee was not required for the current study as participants were not subject to procedures or required to follow rules of behavior which infringed on their physical or psychological integrity [15].

### 2.1. Development of the CSPQ

#### 2.1.1. Identification of FPP Constructs

The instrument was designed to measure FPPs around snacking among Dutch parents of children aged 4–12 years old. We defined FPPs as any behavior of parents that may affect the dietary intake of their child, including both behaviors employed by parents designed to alter child’s dietary intake [6] for example setting food rules, and parental behaviors that might affect the child’s intake but which are not undertaken with that intention, for example providing food when the child is upset. To establish the scope of FPPs constructs, a Delphi study was conducted among experts on this topic [14]. Eighteen constructs were identified by consulting those experts: availability, accessibility, discussing, emotional feeding, instrumental feeding, rules, structure, pressure to eat, providing feedback, permissiveness, encouragement, visibility, rewarding, healthy modeling, educating, involving, meal routines, and monitoring. These constructs were accompanied by specific descriptions of parental behaviors (i.e., 111 descriptions that represent FPPs).

In addition to the Delphi study, twenty qualitative interviews with parents from the target population (five father–mother dyads, fourteen mothers, and one father) were conducted about strategies they use regarding regulating the energy-dense snack food intake of their children. The interviews were semi-structured and held to test if the scope established by experts represent the practices employed among parents. A grounded theory-based method was used to guide the data gathering and analysis [16]. Findings of the interviews clearly indicated that all relevant parenting practices around snacking were identified as no additional FPPs emerged.

The constructs of accessibility, availability, and modelling were each split into two constructs to cover healthy and unhealthy foods. Although our instrument is focusing on snack parenting, parenting practices around healthy foods may influence snack behavior and, based on the interviews, also parents follow this line of reasoning (i.e., trying to reduce unhealthy food intake by stimulating children to eat healthy foods). Differentiating between healthy and unhealthy foods in the measurement of food parenting is also in line with the latest recommendations [17].

#### 2.1.2. Development and Refinement of the CSPQ Items

As we intended to capture the large range of FPPs within one instrument, single-item measures were expected to be advantageous as opposed to multi-item measures, particularly with regard to response burden. An important requirement for single item measurements is that respondents should consider all aspects of the construct of interest [18]. Therefore, all items were accompanied by examples to increase understanding of the construct by parents. Below a global description and the examples (e.g., “parents could have rules concerning eating sweets and snacks for their child. For example, by having the rule that the child should ask first before taking sweets, the child is not allowed to have sweets just before a meal or to have certain sweets and snacks”), the actual question was asked (e.g., “I set rules for [child’s name] about eating EDSFs”) (see Supplementary Materials, File S1). Answering options ranged from completely disagree (1) to completely agree (5) on a five-point scale.

To formulate the 21 questions and accompanying examples, several steps were taken. By using the Delphi study, an initial list of 111 FPPs items was formulated. An online questionnaire survey was used to investigate the occurrence of these 111 behaviors among Dutch parents with children aged 4–12. For this study, 2485 parents from a research panel were invited. The response was 22%, with 524 participants (of which 24.6% fathers) being included in the analyses. Frequency distributions were used to drop items with low variability or extreme means. Ultimately, the 21 CSPQ items and accompanying examples were formulated by selecting those that best reflected the construct of interest, based on the degree of consensus (Delphi study), and those with relevance for the target group (questionnaire survey). The interviews described previously gave us the opportunity to refine items and accompanying examples in line with parents’ phrases. Salient aspects of parental food parenting behaviors, such as specific moments or situations described by parents, are likely to facilitate interpretation by the target population.

Cognitive interviews among six parents from the target population were held to assess a preliminary version of the questionnaire. Verbal proving [19] was used as the primary method to assess if the target group understood the questionnaire as intended, including the introduction, instructions, items, and answering categories. Questions were asked about the entire response process of particular questionnaire items. Example probes include: “what do you think this means?” can you repeat this question in your own words?”, and “how did you arrive at this answer?”. In addition, scientists working in the field of Health Promotion reviewed the instrument to assess face validity (i.e., relevance and comprehensiveness) and wording of items. Both methods led to minor adaptations in the phrasing of a few items and examples. In addition, we decided to tailor each question to the child’s name (e.g., “I have rules for [child’s name] about eating EDSFs”) because we noticed during the cognitive interviews that parents tend to answer questions on their child that elicit the most struggles with regard around eating (e.g., picky eating).

Many experts in the field of parenting, from different countries, and with different backgrounds (e.g., health promotion, pediatrics, and health psychology) participated in our Delphi study. In addition, qualitative interviews with parents were conducted to verify the scope of parenting practices around snacking. By using both studies as a basis for the CSPQ, it is very likely that it contains all relevant FPPs constructs around snacking, contributing to the content validity of the CSPQ.

### 2.2. Test-Retest Reliability

We identified that item ambiguity could be a potential threat to the reliability of the CSPQ. Consequently, respondents might answer items differently on different occasions. Therefore, the questionnaire was assessed on its test-retest reliability [20]. At least 50 respondents were required to finish the second administration of the questionnaire to adequately calculate reliability coefficients [21]. We estimated both response levels (i.e., t1 and t2) to be about 55%, implying that we had to invite 185 parents.

Parents proficient in the Dutch language were recruited at public places in the south of the Netherlands. They were informed about the nature of the study and if interest was expressed, their email address and educational level were asked and gender was reported. A sample of 183 parents from the target population was invited to fill out the questionnaire twice under similar conditions (i.e., self-reported online questionnaire with the same instructions), with a two-week interval. Parents were expected not to change over a period of two weeks when it comes to parenting around snacking while this period would be long enough to prevent recall bias. If parents had more than one child aged 4–12, they were asked to answer the questions about the child who is having his or her birthday first to obtain the most equal distribution in child’s age. This questionnaire also assessed demographic characteristics, including the respondent’s and child’s age and gender and variables to define the respondent’s educational level (i.e., what is the highest level of education you completed?), BMI (i.e., what is your height; what is your weight?), ethnicity (i.e., were you, or (one) of your parents born abroad?) and socio-economic position (SEP; i.e., what is your postal code?), child’s BMI-z [22] and BMI category [23] (i.e., what is your child’s height; what is your child’s weight?).

Predictors of drop out between baseline (t1) and follow-up (t2) were examined using binary logistic regression analysis employing the enter method, with respondent’s age, gender, SEP, ethnicity, BMI and educational level as independent variables. IBM SPSS Statistic 20 (IBM Corporation, Chicago, IL, USA) was used for all statistical procedures.

Test-retest reliability was analyzed by computing intra class correlation coefficients (ICCs) using absolute-agreement and single rating reliability in two-way random models. In addition, the percentages exact agreement between scores on t1 and t2 were calculated for items with poor and fair ICC statistics, as ICC values partly depend on variability in scores [24]. ICCs were classified as poor (≤0.20), fair (0.21–0.40), moderate (0.41–0.60), good (0.61–0.80), and excellent (≥0.81) [25] Agreement scores were classified as poor (<60%), moderate (60–74%), good (75–89%), or excellent (>90%) [26].

## 3. Results

The response on t1 was 60% (*n* = 109), of which 61% completed t2 (*n* = 66). On average, the interval between the two measures was 15.22 days (standard deviation (SD) = 3.29). Female participants were less likely to complete t2 compared to male participants (Odds Ratio (OR) = 2.78, 95% Confidence Interval (CI) 1.07–7.21, *p* = 0.02, Cox and Snell R^2^ = 0.08, Nagelkerke R^2^ = 0.11). Most of the participants were female and highly educated, implying that they are overrepresented (Table 1) [27]. The sample was comparable to the Dutch population with regard to ethnicity and SEP [28,29]. Parents of overweight children were well represented, but the number of underweight children in the sample was higher than national numbers [30]. Focal children were on average seven years (SD = 2.3) of age and parents reported most often on their daughter.

### Test-Retest Reliability Characteristics

ICCs ranged from poor to good (0.23–0.70), with most being classified as moderate (Table 2). Three ICCs were considered to be poor, but the corresponding percentages agreement were found to be acceptable, including discussing (agreement = 60%), rules (agreement = 70%), and availability of healthy foods (agreement = 67%). Five items, referring to the constructs emotional feeding, modeling, visibility, educating, and involving, were found to have good test-retest reliability statistics.

## 4. Discussion

We aimed to develop a food parenting practices instrument for parents with children aged 4–12 measuring the full range of food parenting practice (FPP) constructs and assess its reliability. A questionnaire containing 21 constructs was developed through an extensive process for questionnaire construction and refinement, using a combination of methods. We have shown that the instrument is concise while still covering all pertinent parental behaviors in the context of child snacking.

We found that any item ambiguity had no detrimental impact on reliability as the majority of items showed moderate to good test-retest reliability, indicating its consistency over time. Discussing, rules, and healthy availability had relatively high mean scores and low SDs, which likely explain their unfavorable reliability coefficients (i.e., restriction of range) [31]. Nevertheless, the percentages agreement for these constructs were acceptable. Particularly the item referring to availability of healthy foods might be prone to social desirability bias, which seems to be a common problem for this construct (e.g., [32,33,34]). Vaughn et al. [12] found in their review that 27 of 71 identified validation studies included a test-retest assessment, with generally acceptable correlations. However, in contrast with the current study, authors mostly reported Pearson’s correlations, which are less adequate in test-retest designs compared to ICCs because they do not take systematic error into account [35].

The use of both experts and parents to establish the content and scope of FPPs around snacking resulted in an instrument that likely covers all relevant constructs. This notion is further supported by other studies that aimed at identifying and defining FPPs constructs [17,36,37]. The recently published parenting around Snacking Questionnaire [38] also corroborates many of the constructs measured in the CSPQ. Nevertheless, content validity is a subjective aspect and we invite other scientists to assess it. In the future, a study could be planned in which the CSPQ and P-SNAQ are administered to the same sample of parents to explore strengths and limitations of both.

### 4.1. Applications of the CSPQ

The CSPQ supports the investigation of essential and innovative questions from the research agenda. First, the CSPQ measures a wide range of FPPs, thereby facilitating research to understand how practices are used in combination (e.g., cluster analytic approaches). Those studies are scarce [39], but we already illustrated the usefulness of a person-centered approach by using the CSPQ in a previous paper [40]. It showed that parents use parenting practices in combination, and that this complex interactional nature can be captured perfectly to predict children’s snack intake. Since we believe that the theoretical dimensions of food parenting practices currently proposed in the literature (e.g., autonomy support, coercive control, and structure) [17,36,37] do not fully account for this complex interactional nature of FPPs, we did not test an a priori formulated factor structure (i.e., structural validity) in our ongoing research on measurement properties of the CSPQ. We encourage the use of cluster analyses to further corroborate this notion. Of course, depending on the aims of individual studies, the CSPQ constructs may still be correlated individually or combined to variables of interest. Second, given the instrument’s brevity, response burden is likely to be low. Therefore, a great move forward might also be possible in mediation and moderation research to investigate parenting practices in context [41]. These types of studies involve multiple measures, which is why they would benefit from short measures. Asking too many questions might prevent respondents from completing the questionnaire or lead to drop-out in longitudinal studies with follow-up measures, which is especially problematic in the case of selective drop-out. An example of the application of the CSPQ in the study of contextual factors in the parenting-food intake relationship is also available [42]. In that study, the CSPQ was applied to get a broader view of the relation between FPPs and other concepts, including general parenting. Exploratory factor analyses resulted in four factors: healthy FPPs (6 items, Cronbach’s alpha = 0.85), covert FPPs (4 items, Cronbach’s alpha = 0.67), overt FPPs (2 items, Cronbach’s alpha = 0.70), and non-nutritive FPPs (2 items, Cronbach’s alpha = 0.54). The authors reported that “FPPs and child BMI-z scores were in the expected direction (i.e., healthy FPP were negatively related to BMI z-scores and non-nutritive FPPs were positively related to BMI z-score)” [42] (p. 8).

Since both empirical studies showed relationships between FPPs and child’s outcomes in plausible directions, they provide provisional indications for the CSPQ’s construct validity. In addition, all CSPQ constructs proved to be distinct (i.e., no multi-collinearity) in the two samples, making it feasible to include them into cluster or factor analysis.

### 4.2. Strengths and Limitations

A major strength of the current study is the use of multiple methods to develop the instrument. We determined shortcomings of existing questionnaires and proposed a new questionnaire to forward research in this field. Low educated parents were underrepresented in the test-retest study and female participants were found to be less likely to complete the questionnaire on follow-up, most likely because of motivational aspects and not due to questionnaire weaknesses. Drop-out was about 40%, which is in line with what we expected when designing the test-retest study. On the other hand, it implicates a considerable loss of participants. Probably, participants in the current study were deterred from filling out the questionnaire on t2, because they did not see why it was necessary to fill out the exact same questionnaire twice in such a short time period. Most respondents from our reliability sample were highly educated, resulting in an underrepresentation of low educated parents. This could be seen as a weakness of our study, as it reduces the representativeness of the sample. Against that, testing the reliability of the instrument in a more heterogeneous sample would probably lead to more variation in responses, and thereby to higher reliability statistics [18]. More research is necessary to accumulate evidence on the instrument’s validity.

## 5. Conclusions

The CSPQ covers all relevant food practices related to children’s snacking as currently identified in research among experts and parents. The current study, together with previous work, demonstrated that the instrument is likely to be a feasible tool with acceptable reliability and validity characteristics to map parental food practices. The tool is easy to administer and has low response burden due to the small number of items, which is desirable in studies involving multiple measures (e.g., mediating and moderating processes in research on FPPs). The instrument opens up chances for highly essential research, because it can perfectly be deployed in a cluster analytic approach to gain understanding in the dynamics across a broad range of FPPs.

The current study provides first indications for reliability of the CSPQ. Further studies might be needed to confirm and extend current findings with regard to its measurement properties.

## Figures and Tables

**Table 1:** Descriptive statistics of participants in study.

Participant Characteristics	Reliability Testing (t2 Data)
*n*	66
Age, years (mean [SD])	39.7 (SD = 5.7)
Gender (%)	
Male	42.4
Female	57.6
Educational level (%)	
Low	9.1
Intermediate	30.3
High	60.6
Ethnicity (%)	
Dutch	81.8
Non-Dutch	18.2
BMI	23.9 (SD = 3.0)
BMI category (%)	
Underweight	3.2
Normal weight	64.5
Overweight	32.2
SEP (mean [SD])	0.19 (1.16)
Age of child (mean [SD])	7.4 (SD = 2.3)
Gender of child (%)	
Male	42.4
Female	57.6
BMI child	
Underweight	16.4
Normal weight	72.1
Overweight	11.5

**Table 2:** Test-retest reliability characteristics of the CSPQ (*n* = 66).

FPP Construct	Item ^a^	Mean SD t1 ^b^	Mean SD t2 ^b^	ICC	% Agree
Availability of unhealthy foods	I limit the availability of EDSFs in the house for [child’s name]	3.81 (1.01)	3.76 (0.95)	0.52	
Accessibility of unhealthy foods	I make sure that [child’s name] has easy access to EDSFs	2.61 (1.11)	3.38 (1.04)	0.49	
Discussing	I talk to [child’s name] about eating EDSFs	3.83 (1.01)	3.91 (0.97)	0.23	60
Emotional feeding	I give [child’s name] EDSFs to make [him/her] feel better	1.72 (0.87)	1.91 (0.96)	0.65	
Avoiding unhealthy modelling	I consciously refrain from eating EDSFs when [child’s name] is around	3.17 (1.14)	3.20 (1.13)	0.61	
Instrumental feeding	I give [child’s name] EDSFs to reward him/her for good behavior	2.71 (1.13)	2.48 (1.09)	0.50	
Rules	I have rules for [child’s name] about eating EDSFs	4.43 (0.83)	4.42 (0.66)	0.36	70
Structure	I provide structure regarding EDSFs	3.90 (0.91)	4.08 (0.74)	0.51	
Pressure to eat	I insist that [child’s name] eats or finishes a food item	3.23 (1.23)	3.22 (1.08)	0.58	
Providing feedback	I respond to [child’s name]’s eating behavior by providing him/her with feedback	4.04 (0.74)	4.09 (0.72)	0.50	
Permissiveness	I am flexible about [child’s name]’s eating behavior	2.11 (0.89)	2.25 (1.09)	0.42	
Availability healthy foods	I make sure healthy foods are available at home for [child’s name]	4.48 (0.67)	4.57 (0.56)	0.33	67
Accessibility healthy foods	I make sure [child’s name] has easy access to healthy foods	4.26 (0.82)	4.22 (0.72)	0.48	
Encouragement	I encourage [child’s name] to eat healthy food	4.40 (0.70)	4.42 (0.63)	0.49	
Visibility	I make sure healthy foods are visible for [child’s name]	4.15 (0.89)	4.20 (0.77)	0.61	
Rewarding	I reward [child’s name]’s healthy eating with something else than EDSFs	3.11 (1.11)	3.18 (1.09)	0.41	
Healthy modelling	I intentionally eat healthy foods in front of [child’s name]	3.67 (0.91)	3.65 (1.04)	0.58	
Educating	I teach [child’s name] things about food	4.29 (0.74)	4.28 (0.70)	0.70	
Involving	I involve [child’s name] in food-related activities	4.13 (0.73)	4.09 (0.84)	0.67	
Meal routines	I ensure healthy mealtime habits	4.52 (0.62)	4.46 (0.56)	0.52	
Monitoring	I monitor what [child’s name] is eating during the day	3.63 (1.06)	3.34 (1.09)	0.51	

Note: ^a^ This column shows the actual questions without the global description and examples, each question has been translated into English by a bilingual translator but have not been cross-culturally validated; ^b^ Range from 1 to 5. CSPQ: Comprehensive Snack Parenting Questionnaire; EDSFs: Energy-dense snack foods.

## Supplementary Materials

The following is available online at http://www.mdpi.com/1660-4601/15/5/862/s1, File S1: Comprehensive Snack Parenting Questionnaire (CSPQ).
