# Supplementary material for: The Comprehensive Snack Parenting Questionnaire (CSPQ): Development and Test-Retest Reliability
Source: Int J Environ Res Public Health. 2018 Apr 26;15(5):862. doi: 10.3390/ijerph15050862 (PMC5981901; doi:10.3390/ijerph15050862)
Supplement: Supplementary file 1 [file ijerph-15-00862-s001.pdf]

Supplementary Materials: The Comprehensive Snack Parenting Questionnaire (CSPQ)

Thank you very much for your interest in this questionnaire. This questionnaire is intended for parents of children aged 4–12 years old and is developed by Maastricht University. This questionnaire contains statements about the way you manage your child's eating behaviour.

Many questions refer to energy-dense snack foods. With those foods, we mean all foods that are meant for consumption between meals, such as potato chips, savory snacks, deep-fried snacks, cookies, pastry, sweets, chocolate, and ice cream.

On the following pages, you will see 21 descriptions about how parents may manage their children's eating behavior. Each description is accompanied with several examples. Please answer the questions by indicating if you act this way. The way you act does not have to fully correspond to the examples mentioned.

Please carefully think about [child's name], the child at which the current questionnaire is focusing on.

1. Parents could limit the availability of EDSFs in the house.<br>*For example, by limiting the amount and diversity of EDSFs in the house.*	Strongly disagree	disagree	neither agree nor disagree	Agree	Strongly agree
I limit the availability of EDSFs in the house for [child's name]	○	○	○	○	○
2. Parents could make EDSFs easily accessible to their child.<br>*For example, by storing EDSFs in places where their child can easily get at them.*	Strongly disagree	Disagree	neither agree nor disagree	Agree	Strongly agree
I make sure that [child's name] has easy access to EDSFs.	○	○	○	○	○
3. Parents could talk to their children about EDSF intake.<br>*For example, by talking about the kind of EDSFs their child likes or the kinds that are being bought.*	Strongly disagree	disagree	neither agree nor disagree	Agree	Strongly agree
I talk to [child's name] about eating EDSFs.	○	○	○	○	○
4. Parents could give their child EDSFs to make the child feel better.<br>*For example, by comforting or calming down the child with EDSFs if he/she is in pain or is distressed, or by giving EDSFs to cheer the child up.*	Strongly disagree	disagree	neither agree nor disagree	Agree	Strongly agree
I give [child's name] EDSFs to make [him/her] feel better.	○	○	○	○	○
5. Parents could consciously refrain from eating EDSFs when their child is around.<br>*For example, by eating EDSFs after their child has been put to bed.*	Strongly disagree	disagree	neither agree nor disagree	Agree	Strongly agree
I consciously refrain from eating EDSFs when [child's name] is around.	○	○	○	○	○

6. Parents could give their child EDSFs as a reward for good behavior.<br>*For example, by telling the child he/she has to eat dinner, fruit, or vegetables first before getting EDSFs, or by rewarding the child if he/she has done well (e.g. at school)*	Strongly disagree	disagree	neither agree nor disagree	Agree	Strongly agree
I give [child's name] EDSFs to reward him/her for good behavior.	○	○	○	○	○
7. Parents could set rules for their child about eating EDSFs.<br>*For example, the rule that their child first has to ask for candy before getting it, that their child is not allowed to eat EDSFs just before a meal, or that he/she is not allowed to eat particular types of candy.*	Strongly disagree	disagree	neither agree nor disagree	Agree	Strongly agree
I have rules for [child's name] about eating EDSFs.	○	○	○	○	○
8. Parents could provide structure with regard to EDSFs.<br>*For example, by giving EDSFs only on specific occasions or at fixed times, for example when the child comes home from school.*	Strongly disagree	disagree	neither agree nor disagree	Agree	Strongly agree
I provide structure regarding EDSFs.	○	○	○	○	○
9. Parents could insist that their child eats or finishes a food item<br>*For example, by making their child stay at the dinner table until the plate is empty or by insisting that their child eats fruit.*	Strongly disagree	disagree	neither agree nor disagree	Agree	Strongly agree
I insist that [child's name] eats or finishes a food item	○	○	○	○	○
10. Parents could give their child feedback on his/her eating behavior.<br>*For example, by criticizing their child for eating EDSFs or saying that he/she has had enough EDSFs for the day.*	Strongly disagree	disagree	neither agree nor disagree	Agree	Strongly agree
I respond to [child's name]'s eating behavior by providing him/her with feedback	○	○	○	○	○

11. Parents could be flexible about their child's eating behavior.<br>*For example by setting few limits for the amount of EDSFs their child is allowed to eat.*	Strongly disagree	disagree	neither agree nor disagree	Agree	Strongly agree
I am flexible about [child's name]'s eating behavior	○	○	○	○	○
12. Parents could make sure that healthy foods are available for their child at home.<br>*For example, by bringing fruit and vegetables home or by keeping a variety of healthy foods for their child.*	Strongly disagree	disagree	neither agree nor disagree	Agree	Strongly agree
I make sure healthy foods are available at home for [child's name]	○	○	○	○	○
13. Parents could make sure their child has easy access to healthy foods.<br>*For example by storing healthy foods in a place that is easily accessible to their child, or by having healthy foods, such as fruit, available in ready-to-eat form.*	Strongly disagree	disagree	neither agree nor disagree	Agree	Strongly agree
I make sure [child's name] has easy access to healthy foods	○	○	○	○	○
14. Parents could encourage their child to eat healthy food.<br>*For example, by encouraging their child to eat enough vegetables during the meal, by being positive about healthy foods, or by encouraging their child to eat a variety of foods.*	Strongly disagree	disagree	neither agree nor disagree	Agree	Strongly agree
I encourage [child's name] to eat healthy food	○	○	○	○	○
15. Parents could make sure healthy foods are visible for their child.<br>*For example by putting a fruit bowl in a place where the child can clearly see it.*	Strongly disagree	disagree	neither agree nor disagree	Agree	Strongly agree
I make sure healthy foods are visible for [child's name]	○	○	○	○	○
16. Parents could reward their child for healthy eating behavior (rewarding with something else than EDSFs).<br>*For example by complimenting their child on eating healthy food or by giving their child a sticker for eating healthy food.*	Strongly disagree	disagree	neither agree nor disagree	Agree	Strongly agree
I reward [child's name]'s healthy eating with something else than EDSFs	○	○	○	○	○

17. Parents could intentionally eat healthy foods in front of their child.<br>*For example by eating fruit when their child is around or by talking enthusiastically about healthy foods when their child is around.*	Strongly disagree	disagree	neither agree nor disagree	Agree	Strongly agree
I intentionally eat healthy foods in front of [child's name]	○	○	○	○	○
18. Parents could teach their child about food.<br>*For example, by explaining that eating healthy food is better than eating EDSFs or that eating EDSFs is bad for their teeth or that they might become overweight by eating EDSFs.*	Strongly disagree	disagree	neither agree nor disagree	Agree	Strongly agree
I teach [child's name] things about food	○	○	○	○	○
19. Parents could involve their child in food-related activities.<br>*For example, by taking their child along when doing the groceries, letting them choose what to have for dinner, or letting them assist with preparing the food or setting the table.*	Strongly disagree	disagree	neither agree nor disagree	Agree	Strongly agree
I involve [child's name] in food-related activities	○	○	○	○	○
20. Parents could ensure healthy mealtime habits.<br>*For example by having meals together with the whole family or by eating at the dinner table.*	Strongly disagree	disagree	neither agree nor disagree	Agree	Strongly agree
I ensure healthy mealtime habits	○	○	○	○	○
21. Parents could monitor what their child is eating during the day.<br>*For example, by checking how much EDSFs their child has eaten or by asking their child what he or she ate.*	Strongly disagree	disagree	neither agree nor disagree	Agree	Strongly agree
I monitor what [child's name] is eating during the day	○	○	○	○	○

Note: the questionnaire has been translated by a bilingual translator but has not been cross-culturally validated.
